# Ecological niche modeling of rabies in the changing Arctic of Alaska

**DOI:** 10.1186/s13028-017-0285-0

**Published:** 2017-03-20

**Authors:** Falk Huettmann, Emily Elizabeth Magnuson, Karsten Hueffer

**Affiliations:** 1 EWHALE Lab, Institute of Arctic Biology, Department of Wildlife Biology, University of Alaska Fairbanks, 902 N. Koyukuk Dr., P.O. Box 757000, Fairbanks, AK 99775 USA; 2 Department of Biology and Wildlife, University of Alaska Fairbanks, 982 N. Koyukuk Dr., PO Box 756100, Fairbanks, AK 99775 USA; 3 Department of Veterinary Medicine, University of Alaska Fairbanks, 901 Koyukuk Drive, PO Box 757750, Fairbanks, AK 99775 USA

**Keywords:** Rabies, Alaska, Ecologic niche, Data mining, Predictions

## Abstract

**Background:**

Rabies is a disease of global significance including in the circumpolar Arctic. In Alaska enzootic rabies persist in northern and western coastal areas. Only sporadic cases have occurred in areas outside of the regions considered enzootic for the virus, such as the interior of the state and urbanized regions.

**Results:**

Here we examine the distribution of diagnosed rabies cases in Alaska, explicit in space and time. We use a geographic information system (GIS), 20 environmental data layers and provide a quantitative non-parsimonious estimate of the predicted ecological niche, based on data mining, machine learning and open access data. We identify ecological correlates and possible drivers that determine the ecological niche of rabies virus in Alaska. More specifically, our models show that rabies cases are closely associated with human infrastructure, and reveal an ecological niche in remote northern wilderness areas. Furthermore a model utilizing climate modeling suggests a reduction of the current ecological niche for detection of rabies virus in Alaska, a state that is disproportionately affected by a changing climate.

**Conclusions:**

Our results may help to better inform public health decisions in the future and guide further studies on individual drivers of rabies distribution in the Arctic.

**Electronic supplementary material:**

The online version of this article (doi:10.1186/s13028-017-0285-0) contains supplementary material, which is available to authorized users.

## Background

Rabies is a global zoonotic disease that lacks satisfactory treatment and kills 50,000–70,000 people annually, mostly in developing countries where dog-associated rabies is not well controlled [1]. In developed countries rabies among wild animals poses a threat to human health through direct contact with infected wildlife or through the infection of unvaccinated dogs, and cats [2]. The economic burden of rabies is significant even in areas without large numbers of human rabies cases due to the costs of prevention efforts and required infrastructure [1].

In the circumpolar region the arctic fox (*Vulpes lagopus)* is considered the primary maintenance host for rabies [3]. The arctic fox has been displaced in some regions by the red fox (*Vulpes vulpes*) presumably driven by anthropogenic change [4–6]. However, this trend is not found in all regions of the Arctic [7].

In Alaska, rabies is of significant concern to public health, particularly in the face of environmental change [8], see also Additional file 1 for detail on human health implications. Enzootic rabies (defined as always being present at a certain level) is believed to be primarily limited to northern and western coastal regions of Alaska that have only limited human development [9]. Occasionally epizootic rabies occurs in interior regions of Alaska [10]. Although the exact extent of enzootic regions is unknown. Large urban settlements such as the cities of Anchorage, Fairbanks and Juneau, are not directly affected by enzootic rabies apart from occasional importation of the disease through translocation of infected dogs from enzootic rural areas (for an example see [11]). The regions of Alaska with the highest burden of rabies cases in both wildlife and domestic dogs, like many other remote arctic communities, generally lack adequate veterinary care and dog vaccination. In addition, the true burden of rabies, especially in foxes is not known, because diagnostic testing is generally limited to incidents of possible human exposure and animals suspected of having rabies in regions considered non-enzootic. There is little active surveillance of rabies among wildlife in enzootic regions of Alaska. The majority of rabies testing occurs only in close proximity to human infrastructure. Industrial developments in remote areas are known to enhance invasive species, including diseases (see [12] for invasive species in Alaska) and can provide significant attractions to wildlife through food subsidies, as well as olfactory or light stimuli [13, 14].

**Fig. 1:**
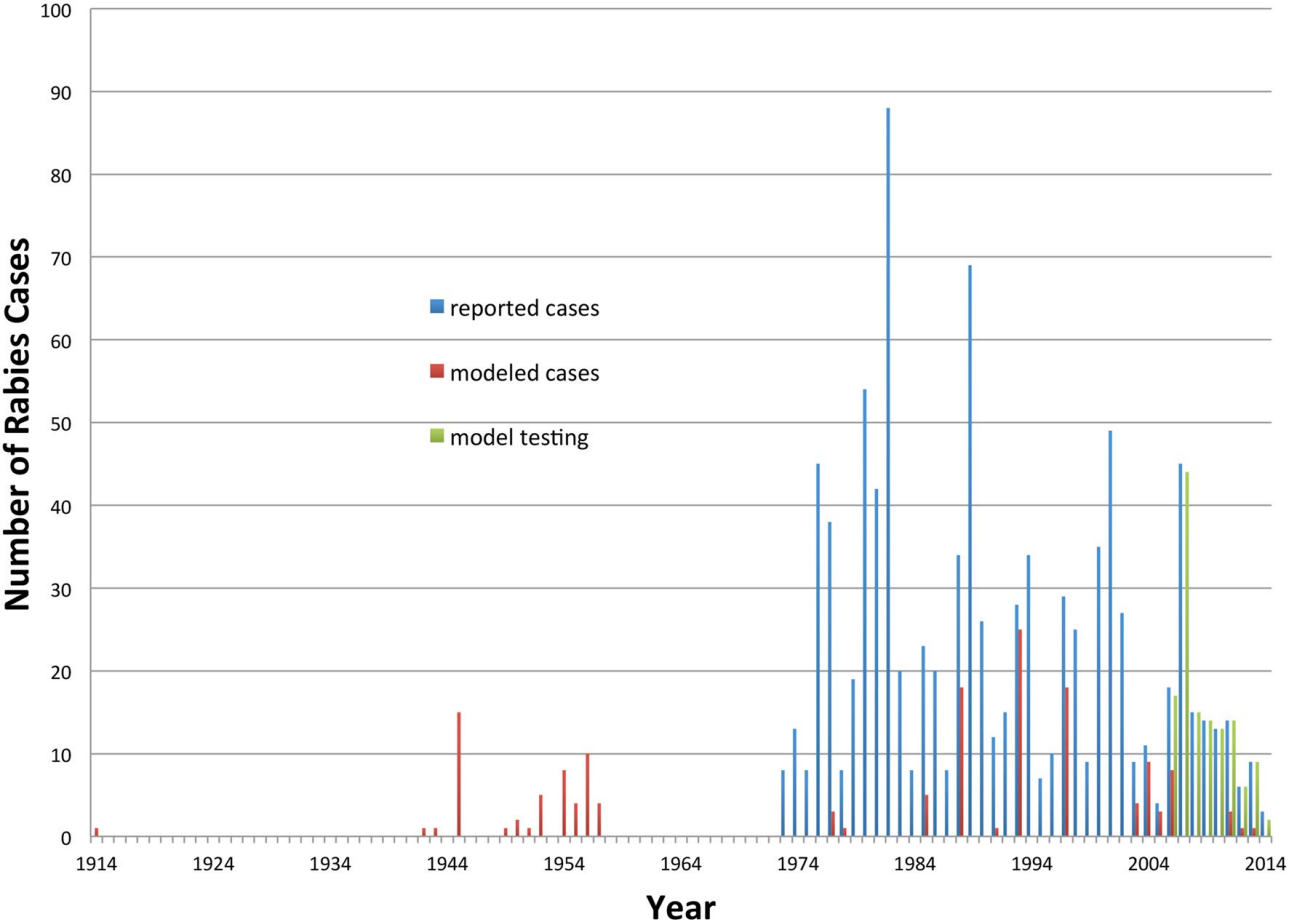
Diagnosed rabies cases over time in animals and people. *Blue bars* represents reported cases according to the annual infectious disease reports (1973–2014) published by the Section of Epidemiology for the State of Alaska. The *red bar* represents cases used to train our models (Additional file 2) and the *green bars* represents the cases included in testing our models (Additional file 3)

Rabies dynamics in Alaska are characterized by cyclical increases in reported cases with 4–5 year intervals [15] (Fig. 1). During the period from 2000 to 2014, 272 animals were reported positive for rabies by the Section of Epidemiology for the State of Alaska in their annual disease reports [16–23]. Ninety-nine percent of these rabies-positive animals originated in Northern and Southwestern Alaska that are considered enzootic for wildlife rabies. In contrast South-central and parts of central interior Alaska did not contribute any cases of rabies in terrestrial mammals. The spread of arctic variant rabies into areas previously not affected poses a risk even in the more populated areas of Alaska. This can be seen by the spread of arctic variant-rabies into southern Ontario for instance [24].

Both red and arctic foxes are frequently diagnosed with rabies, but red foxes are diagnosed with rabies more often than arctic foxes [15]. Within Alaska the rabies virus is maintained as three distinct genetic variants [25, 26]: Arctic rabies variants 2, 3 and 4. The general spatial distribution of these variants seems to be stable [25–27]. The biogeography and mechanism of maintaining at least three distinct strains over time is not well understood [27]. However, the population structure of arctic foxes appears to be more closely related to the distribution of rabies variants compared to the population structure of red foxes. It suggests that the mesocarnivore arctic fox is the maintenance host, while the red fox serves as a frequent spillover host for this virus. Alternatively, the red and arctic fox provide a dynamic multi-host maintenance system for arctic rabies virus variants in Alaska [27]. The consequences on rabies dynamics of a supposedly increased displacement of arctic foxes by red foxes is not known [28]. However Kutz provides examples for increased disease in Northern regions, mainly parasitic infections, associated with extreme weather events and warmer temperatures [29]. Similar dynamics could also hold true for rabies at high latitudes.

Some examples of increased disease transmission in the circumpolar North due to a changing climate have been described [30]. With climate change predicted to be more extreme at high latitudes, e.g. 10 or more degrees Celsius temperature increase during the next 100 years [31], it is imperative to base future public health decisions on the best available data and predictions [32]. This should be guided by public access, transparency, repeatability, as well as a thorough and justifiable understanding of the ecological niche occupied by the disease of concern [33].

Because of a sampling effort bias towards human development and under-sampling of animals for rabies diagnostics from remote areas, a complete picture of the presence and prevalence of rabies does not yet exist for Alaska. To overcome such problems, predictive modeling emerged as a powerful method, based on empirical data and best-available science ([34] for rabies; for other examples see [35–38]). Organisms, including pathogens and their hosts, are bound by a certain ecological niche [32, 33, 39]. Describing and predicting the ecological niche of a disease can greatly help to further our understanding of pathogen dynamics, even in the face of limited sampling [40, 41].

Following best practice and state-of-the art methods [33, 34, 38, 41, 42], this investigation tried to define the quantitative envelope of the ecological niche for rabies in the Arctic using Alaska as a test case. We carried out such an analysis with an ecological niche model using machine learning algorithms, based on geographical information systems (GIS) and publicly available environmental data, applied to presence only locations of compiled rabies detections.

## Methods

Publically available information on 153 diagnosed rabies cases from 1914 to 2013, in terrestrial mammals was compiled and manually divided into a stratum that occurred in areas considered enzootic by the State of Alaska Section of Epidemiology, and a second stratum diagnosed outside this enzootic area [9] (Additional file 2). The classification of enzootic or non-enzootic greatly influences rabies control measures. An independent set of recent diagnosed rabies cases (Additional file 3) was used to compare different approaches.

Rabies cases were model-predicted with machine learning algorithms comparing them to pseudo-absences (created randomly in GIS for Alaska). Classification and regression trees (CARTs)- based boosting and bagging (TreeNet, RandomForest, SPM7, Salford Systems Ltd) using the ‘default’ settings for those models because they are specifically designed for presence data, data mining (see Table 1 for details) were used to model the ecological niche of rabies in Alaska. These model settings generalize best for data such as used here (https://www.salford-systems.com/products/treenet) [33, 35]. Because these models employ ‘recursive partitioning’ the models are rather robust for correlations and interactions, as judged by high AUC ROCs and assessment metrics [33].

**Table 1:** Settings and explanations of the TreeNet model run

Metric	Setting	Effect	Justification
Learnrate	AUTO	A detailed but slow model run	Known to provide best results for the algorithm ‘learning’ data
Subsample fraction	50%	Internal testing while model is grown	Standard approach for balanced tree models
Logistic residual trim fraction	0.10	Fine-tuning	Allows for better fits
Huber-M fraction of error squared	0.90	Accuracy level	A statistical standard threshold for certainty
Optimal logistic model selection	Cross entropy	How to find the optimal model	Usually the best setting for tree-based models
Number of trees to build	1000	Number of trees tried out for the best solution	This number should widely overshot the known optimum
Maximum number of nodes	6	Determines the node depth of trees used	This number determines whether a ‘stump’ or a fully fit tree is run
Terminal node minimum training cases	10	For most data cases it provides a robust tree	Number of cases for each tree branch split
Maximum number of most-optimal models to save summary results	1	Just 1 most-optimal model is saved	
Regression loss criterion	Huber-M (Blend LS and LAD)	A statistical metric to express gain vs cost of a new rule	Standard approach in trees

The environmental layers used are shown in Table 2. These model layers are known to contribute to the ecological niche, and also act as a proxy to inquire further if deemed relevant in future studies. In addition, these layers are currently ‘the best available GIS layers for the state of Alaska [35, 43, 44].

**Table 2:** Predictors of rabies in Alaska and for assembling the ecological niche

Predictor	Source	Comment
Euclidean distance to Alaska coastline	Alaska GAP data	Obtained with ArcGIS tools
Euclidean distance to Alaska infrastructure	Alaska GAP data	Obtained with ArcGIS tools
Elevation	Alaska GAP data	
Monthly mean temperature	Alaska GAP data (taken from SNAP)	
Monthly mean precipitation	Alaska (taken from SNAP)	

For public data sources see [43, 44]

For improved inference and validity, models should be assessed for their predictive performance in order to express their reliability [33, 40]. AUC ROC inherent in Salford Predictive Modeler (SPM) was one performance metric used. Machine learning approaches, as used in this study, express the ecological niche as a relative index of occurrence (RIO) visualized in the figures along a quantitative (color) gradient, red-yellow-green. Red is essentially high RIO, yellow is a mid range value, and green is low RIO.

Finally, in order to better predict the distribution of rabies in Alaska for the future, the climate niche models of rabies was predicted to 2050, using regionalized IPPC climate models for Alaska. Predictors for this model of a possible future rabies niche are limited to climate ones because Alaska still lacks reliable and available planning scenarios for the future explicit in space and time for land cover and its socio-economic features [45, 46]. 2050 was used as a more realistic and testable ‘future’, and thus having a real-world application.

## Results

**Fig. 2:**
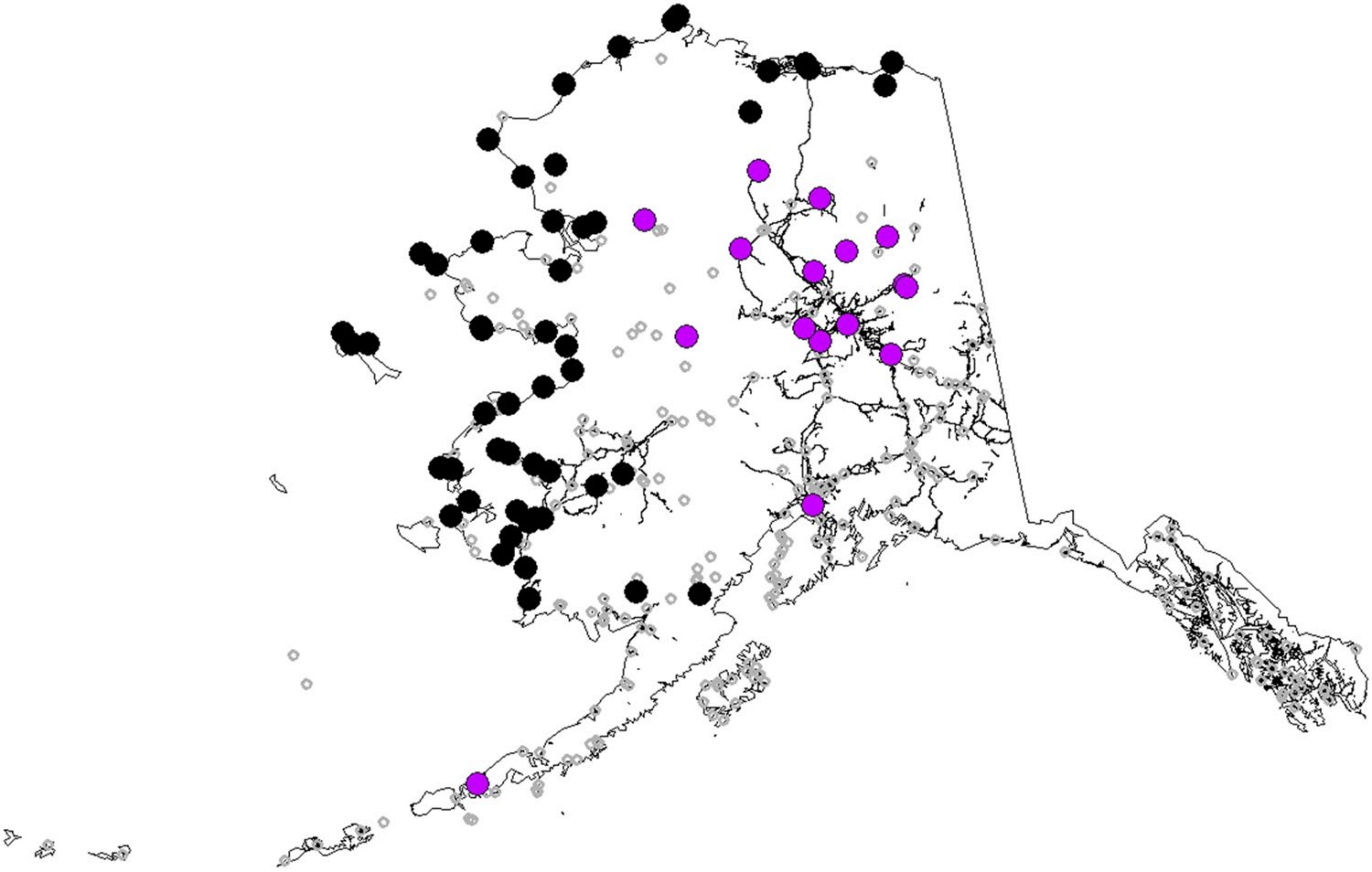
Alaska map and location of diagnosed rabies cases used to build models. Cases classified as enzootic is indicated in *black* and epizootic cases in *purple*. Settlements and road infrastructure is shown in *grey*

This study provides for the first time publically available data of 153 confirmed rabies cases from 1914 to 2013 with different degrees of geo-referencing quality. This data set is available in Additional file 2 and from the authors upon request (sensu Zuckerberg [47]). This dataset is an essential part of the result. The cases of terrestrial rabies (excluding 2 bat cases) were divided into two subsets: confirmed animal rabies cases from the area of Alaska considered enzootic for rabies, and areas not considered enzootic (Fig. 2). The latter cases were considered associated with sporadic epizootics. Most of these epizootic associated cases were temporally associated with a large-scale outbreak in interior Alaska during the 1950s [10]. Using these data sets machine learning algorithms were utilized to build the following three ecological niche models each for a test which provides us the best generalization for Alaska: models were informed by (a) only cases from areas considered enzootic for rabies (enzootic cases), (b) only cases from non-enzootic areas (outbreak cases), and (c) all confirmed rabies cases. Utilizing these three approaches models were created and assessed for performance, and then predicted risk maps for rabies detection in Alaska were generated. ‘Risk’ is defined here as pixels with a relative index of occurrence of rabies, as predicted from the model [35, 41].

These maps of the relative index of occurrence varied somewhat, depending on the capability of the algorithm employed and on the data used to inform the model. However, all models predicted the northern coastal areas as high-risk areas for the detection of rabies, which is even true for models only informed by outbreak-associated samples, which excluded samples from this area. Another area consistently identified among all models is located south of the Brooks Range east of Chandalar Lake (Eastern Yukon River Basin). This area is of interest because cases from that region were not included in the data set that informed the model based on enzootic cases. However, this area was involved in the outbreak in the middle of the twentieth century [10] and it has recently seen isolated cases of rabies at its western most boundary [48].

**Fig. 3:**
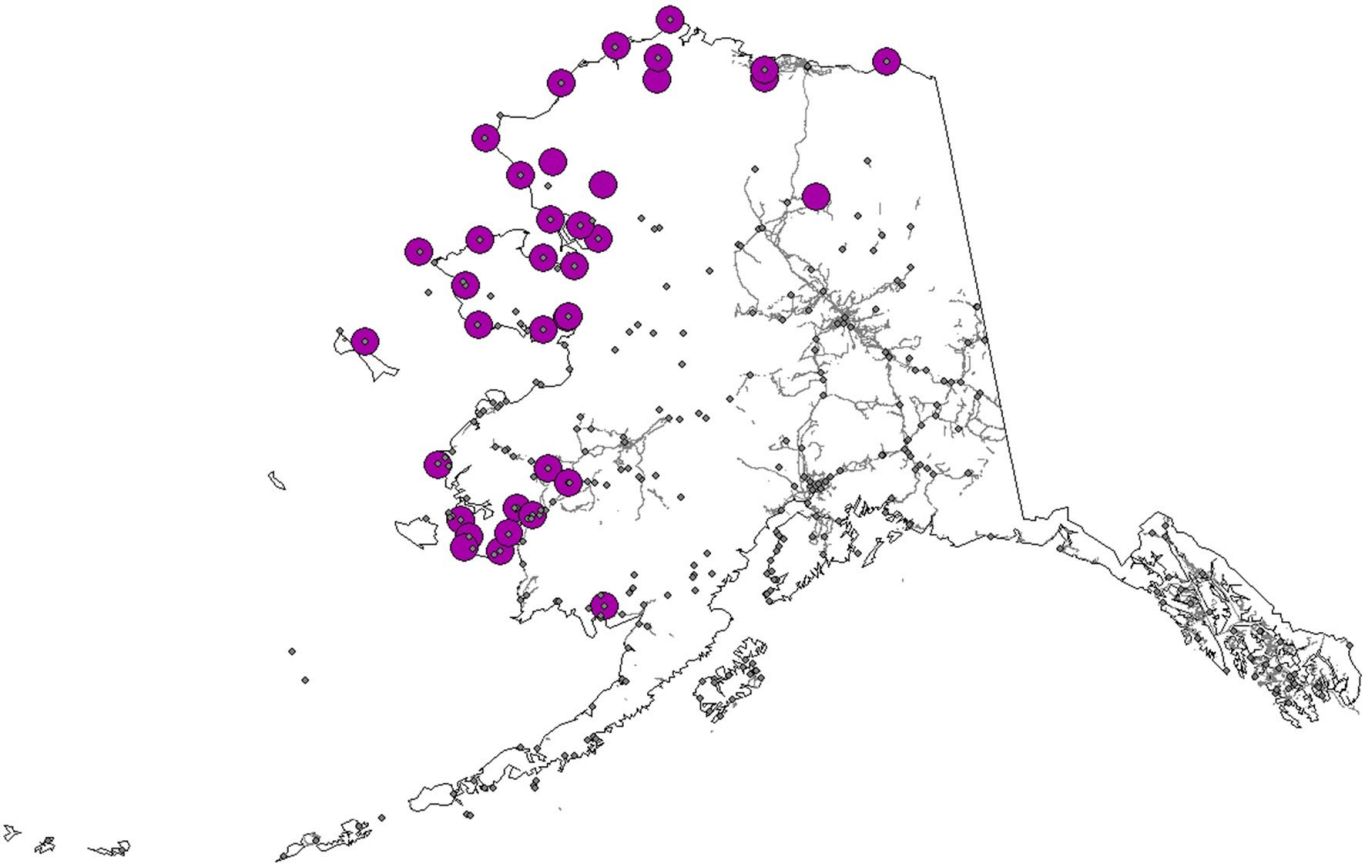
Alaska map and location of diagnosed rabies cases data to assess model performance. Seventy three locations were used, representing 127 diagnosed cases to assess the models

**Fig. 4:**
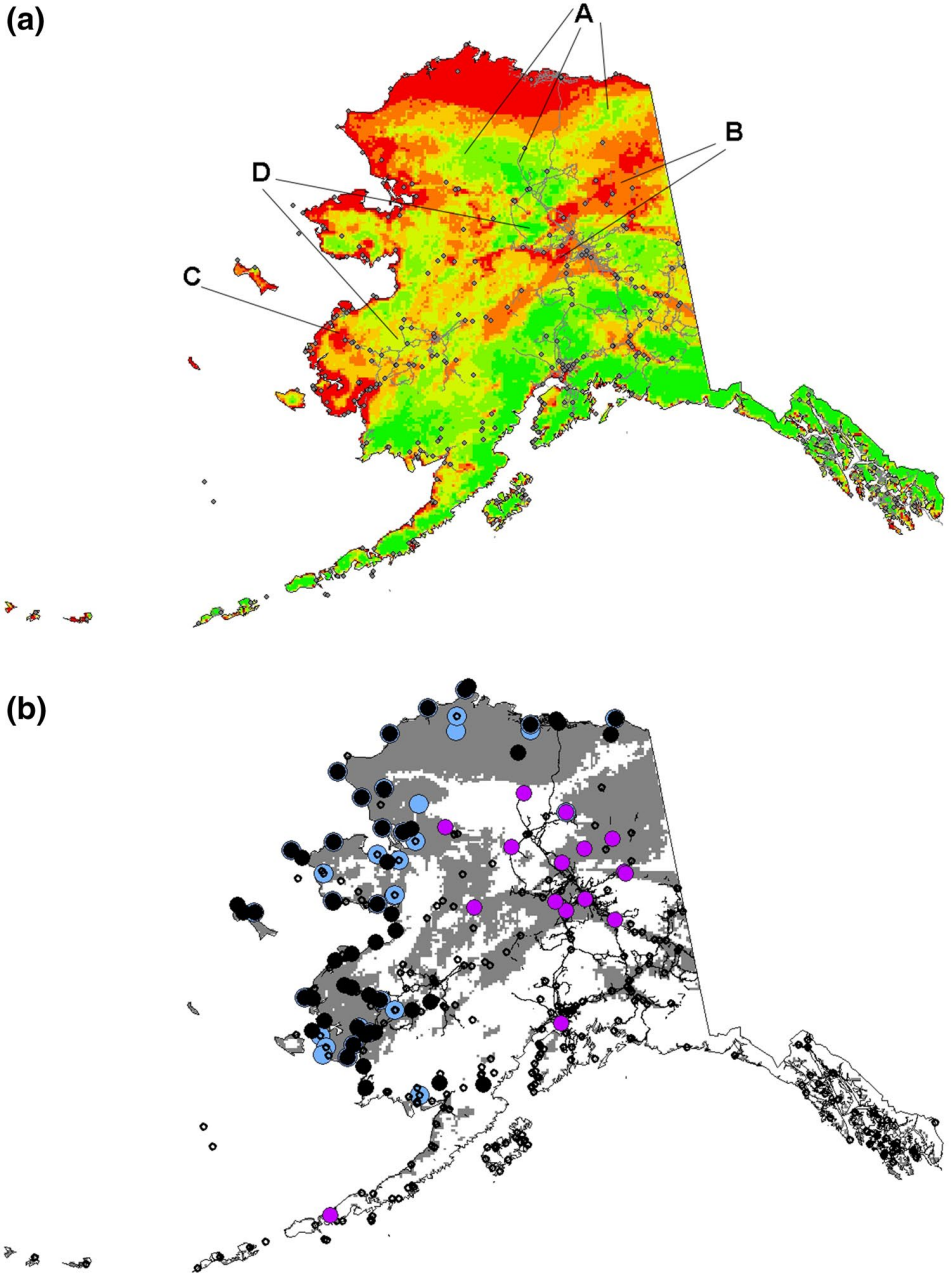
**a** Best TreeNet model (pooled data) prediction of rabies in Alaska. *Colors* show relative index of occurrence (RIO), where *red* is high RIO, *yellow* is mid range RIO and *green* is low RIO; rabies used to build the model are overlaid for overview. *Letter* indicate regions of special interest in the model output: *A* Brooks Range, *B* Eastern Yukon Basin, *C* Lower Yukon/Yukon Delta, *D* Middle Yukon. **b** The same RIO map classified into a presence/absence scheme. Rabies cases used to build the model are indicated in *black* and *purple*; (see Fig. 1a) and assessment data in *blue*) are overlaid for overview

To better compare the different approaches, the models were confronted with a compiled set of recent rabies cases detected by the Alaska State Public Health Laboratory (Fig. 3). The model based on the TreeNet algorithm and informed by all available rabies cases in our data set performed best (Fig. 4; Additional file 4). The remainder of the result section will therefore focus on this model for inference.

This TreeNet-based model identified large areas north of the Brooks Range and areas south along the coast into the Yukon Kuskokwim Delta as areas at highest risk for rabies detection in the state. Interestingly, while the Eastern Yukon River Basin was identified as a high-risk area for rabies and the mouth of that river is also identified with the high-risk area to the West, the middle section of this major river in Alaska was not identified as an area of high probability for rabies detection. Terrestrial rabies is widely predicted to be absent in southern Alaska, except for the major population center of Anchorage.

The best performing model identified distance to infrastructure, elevation, distance to coast, precipitation in June, and precipitation in February as predictors most important in defining the ecological niche (Table 3).

**Table 3:** TreeNet variable importance of parameters utilized in best performing model (148 Alaska rabies data locations pooled regardless of outbreak or enzootic locations)

Variable	Score
Distance to infrastructure	100.00
Elevation	56.09
Distance to coast	31.95
Precipitation June	30.63
Precipitation February	21.78
Precipitation October	20.28
Temperature October	20.27
Precipitation March	19.22
Precipitation May	18.64
Temperature April	18.49
Precipitation August	18.29
Temperature December	17.68
Temperature February	17.56
Precipitation April	16.89
Precipitation July	16.77
Precipitation September	16.47
Precipitation December	14.99
Precipitation January	13.40
Temperature August	13.13
Temperature November	12.93
Temperature January	12.50
Temperature May	11.64
Temperature March	10.25
Precipitation November	9.44
Temperature September	8.89
Temperature June	8.64
Temperature July	5.62

The variables are listed by importance together with their relative score in informing the model on the likelihood of rabies occurrence

A model built in TreeNet using only climate variables had a lower performance than the model build on all predictors (namely the human infrastructure ones). However, it repeated the general results, also identifying similar areas of the state with some extended areas in the Yukon-Kuskokwim Delta compared to a model including non-climate variables.

**Fig. 5:**
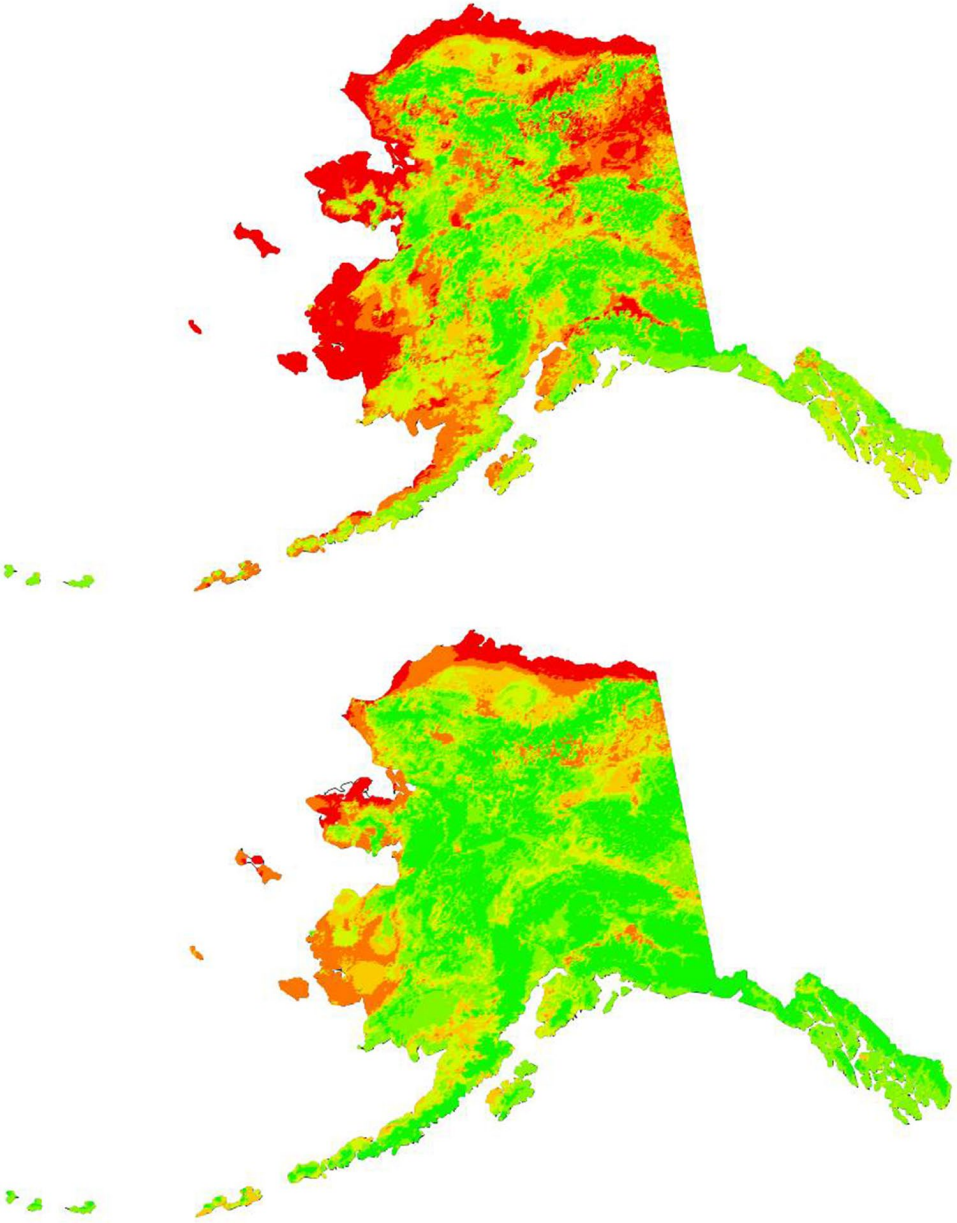
Climate niche predictions of rabies using Treenet. The *top panel* shows the rabies prediction using the climate niche from 2010 [A1B1 obtained from scenarios network for Alaska + Arctic planning (SNAP)]. The *bottom panel* depicts the rabies prediction using the climate niche from 2050 (A1B1 obtained from SNAP)

Unfortunately, we lack any reliable planning and forecast maps and models of infrastructure for Alaska. While those exist for many climate variables [46] they are not available for future development of human infrastructure. We therefore utilized only this climate-based ecological niche model for starting to explore the possible effects of climate change, such as warming in the Arctic and altered precipitation, on the rabies risk distribution in Alaska for the predicted climate scenario in 2050. As done elsewhere [14], we employed an ecological niche model projecting the climate-based niche onto climate data predicted for the year 2050 using the regionalized IPCC climate model from SNAP (A1B1 scenario). This resulted in a significantly reduced area of predicted future risk of rabies detection, especially in the southern areas of current rabies risk prediction (Fig. 5).

## Discussion

Disease prediction is a common effort that can increase understanding of disease ecologies, especially in remote areas [32, 35, 41, 49, 50]. Our approach to better understand rabies dynamics in the circumpolar region becomes possible due to publically available and shared data of confirmed rabies cases, as well as environmental GIS layer predictions and non-parsimonious algorithms. This modeling effort identified several geographic areas of predicted risk for rabies detection. Further, variables were identified by our modeling approach that influenced the distribution of rabies detection throughout the State, specifically the relevance of human infrastructure. A major limitation of our modeling approach was the way most of the data informing the model were collected. Rabies testing in Alaska is largely performed by the public health system with a focus, and consequent bias, towards human exposures. Vast areas in Alaska such as wilderness areas remain largely unstudied for wildlife diseases including rabies. Because of this, knowledge of rabies distribution and ecology Alaska is rather poor and biased through a human-focused detection system. The current pragmatic focus on possible human exposure could skew our model towards ignoring the true role of areas further away from human infrastructure as a variable responsible for majorly influencing the predicted presence of wildlife rabies. However, if one considers our models as an approach to determine possible risk for humans to encounter the rabies virus, this possible bias will still be very reflective of a threat to human health. On the other hand, this bias is likely leading to an underestimation of rabies cases in Alaska. It is still limiting our ability to identify additional variables influencing rabies distribution in remote areas that are relatively unaffected by human activity. Arguably, one wants to know and use as many predictors as possible to test and describe rabies outbreaks, instead of just a parsimonious one.

Our modeling approach provides predictions explicit in space and time and does not attempt to elucidate direct causal relationships between identified predictors and rabies risk. For example, the identified climate variables likely influence rabies occurrence indirectly through effects on wildlife populations rather than direct effect on virus particles or replication of the virus. However, identifying these predictors without detailed knowledge on mechanisms is still important to describe the niche and help focus public health efforts in a spatially explicit form. Large uninhabited areas of Alaska within or adjacent to areas considered enzootic for rabies virus are not systematically surveyed. This limits our ability to fully understand the ecological drivers of this important disease. In addition, information on possible variables at an appropriate landscape level, such as density of reservoir and spillover hosts is needed to better model the ecological drivers of rabies distribution in Alaska. An additional limitation is the possible misdiagnosis of other diseases (such as canine distemper in foxes) as rabies, especially for cases in the early stages of disease. However, as these cases follow a similar pattern to more recent cases we see this as a minor limitation only.

Our rabies forecast for the state into the future using climate models for 2050 shows a decay of the Arctic rabies niche for the arctic rabies variants. However, we currently lack any information on how rabies variants from the south could enter the state and how they could behave and disperse in a warming Arctic. In addition, the adaptation of the arctic rabies virus variants to a changing environment and host distribution warrants caution in overly relying on our prediction of the extent of the ecological niche for just this rabies virus variant into the future. Our finding that human infrastructure possibly plays a central role, and assuming an increase of infrastructure development, casts doubt on our prediction of reduced rabies risk in a changing Alaska.

Despite the limitations mentioned above, the modeling approach and the results presented can still help public health officials to better focus preventative efforts in the areas most at risk of rabies exposure to humans. Such efforts could include traditional measures such as possible active surveillance efforts in predicted hotspots and coldspots, increased dog vaccinations and population controls and vigilance to detect possible outbreaks or expansion of enzootic areas in the face of a changing Arctic. While currently licensed oral vaccines have been shown to be effective in protecting arctic foxes against infection with virus circulating in Alaska [51], large-scale use of these measures to control rabies are unlikely to be cost effective [51]. However, our methods, open access compilation and results might guide a more limited use of this intervention tool.

Our modeling can especially help target active surveillance efforts in less developed areas of the state. These efforts could test the model presented here and greatly advance our understanding of relevant drivers of rabies maintenance in pristine Arctic areas.

In future work this model and template should be tested and applied further with independent data, ideally data that is less biased and not dependent on human access and human exposure. We also believe that a wider macro-ecology view and model prediction for rabies overall, and its niche is warranted, assuming that other rabies strains from Canada or more southern regions will enter Alaska sooner or later. This pathogen transport has been seen in other disease system with influenza being a prominent example of pathogen transport to high latitudes [52]. A wider socio-economic perspective to public health and rabies across scales is required. Such an approach will clarify how the findings of our model can be extended beyond the risk of human exposure to start to explain and manage the distribution of rabies in Alaskan wildlife.

## Conclusions

I this paper we showed that machine learning approaches and open data sources can help predict the ecological niche of infections disease detection for an important zoonotic disease in the Arctic. These findings can help guide future surveillance efforts as well as inform public health officials in focusing efforts on areas at high risk for rabies virus infections. Future work should test our modeled predictions and lead to further refinement of our predicted ecological niche of rabies virus in Alaska.

## Additional files

**Additional file 1.** Public health impact of rabies in Alaska. This file contains information on the historic public heath impact of rabies in Alaska to put our findings into an appropriate context of One Health.

**Additional file 2.** Locations of rabies cases in Alaska 1914–2013 used in model development. This file contains the location, dates and vectors of all rabies cases used to build our models. In addition the files indicates if a case was considered part of enzootic rabies or associate with an outbreak in a non-enzootic area.

**Additional file 3.** Location data of rabies cases used for model assessment. This file lists the location of rabies cases provided by the Alaska Section of Epidemiology in the Department of Health and Social Services of the State of Alaska.

**Additional file 4.** TreeNet model summary statistics for pooled rabies locations. This file contains summary statistics for the best performing model developed in this studies. It includes partial dependence plots for the three most important predictors in the model (distance to infrastructure, Elevation, and distance to coast), genral Average likelihood statistics and a Gains chart for 104 trees.
