# Supplementary material for: Ecological niche modeling of rabies in the changing Arctic of Alaska
Source: Acta Vet Scand. 2017 Mar 20;59:18. doi: 10.1186/s13028-017-0285-0 (PMC5359834; doi:10.1186/s13028-017-0285-0)
Supplement: Supplementary file 1 — Additional file 1. Public health impact of rabies in Alaska. This file contains information on the historic public heath impact of rabies in Alaska to put our findings into an appropriate context of One Health. [file 13028_2017_285_MOESM1_ESM.pdf]

Additional file 1. Public health impact of rabies in Alaska

During the period 2002-2012, 148 humans received post-exposure prophylaxis (PEP) for possible exposure to rabid animals in Alaska [1,2]. The majority of these cases were exposed in areas considered enzootic for rabies (from 2008-2012 all PEP were administered due to exposure in enzootic areas). The majority of animals known to be involved in exposures in Alaska were dogs [1]. Rates of serious dog bites to humans (those requiring hospitalization) are significantly higher in Alaska than the national average. The high rate of those severe dog bite injuries are concentrated in the same regions as reported cases of animal rabies on a per capita basis [3] This highlights the importance of rabies to human health in Alaska, especially in those regions with enzootic rabies activity that are characterized by significant healthcare disparities and limited access to both veterinary and human medical services [4].

References

1. Castrodale, L., 2009: Use of rabies postexposure prophylaxis supplied by the Alaska Section of Epidemiology, Alaska, 2002-2007. Public Health Reports 2009; 124:262–266.

2. Alaska Rabies Post-Exposure Prophylaxis: 2008–2012 Data Summary and Policy Change. Bulletin No. 19 Alaska State Section of Epidemiology, 2013. http://epibulletins.dhss.alaska.gov/Document/Display?DocumentId=85 Accessed on 14 October 2016 .

3. Dog Bite Injury Hospitalizations and Estimated Burden in Alaska, 2001–2011. Bulletin No. 29 2015. Alaska State Section of Epidemiology, 2015. http://epibulletins.dhss.alaska.gov/Document/Display?DocumentId=10 Accessed on 14 October 2016.

4. Young TK, Rawat R, Dallmann W, Chatwood S, Bjerregaard P. Circumpolar Health Atlas. University of Toronto Press, Canada; 2012.
