# Supplementary material for: Ecological niche modeling of rabies in the changing Arctic of Alaska
Source: Acta Vet Scand. 2017 Mar 20;59:18. doi: 10.1186/s13028-017-0285-0 (PMC5359834; doi:10.1186/s13028-017-0285-0)
Supplement: Supplementary file 2 — Additional file 2. Locations of rabies cases in Alaska 1914–2013 used in model development. This file contains the location, dates and vectors of all rabies cases used to build our models. In addition the files indicates if a case was considered part of enzootic rabies or associate with an outbreak in a non-enzootic area. [file 13028_2017_285_MOESM2_ESM.pdf]

Additional file 2. Locations of rabies cases in Alaska 1914 - 2013 used in model development

LAT	LONGDEC	DATE	VECTOR	RabOutbrk
65.913000	161.924000	Feb. 1914	Dog	1
66.837222	161.036667	Jan. 1942	Wolf	1
70.647222	160.016111	1943	Wolf	1
65.533333	144.695556	Oct. 1945	Red Fox	0
65.533333	144.695556	Dec. 1945	Wolf	0
65.483333	144.634167	1945	Red Fox	0
64.737222	155.487778	1945	Red Fox	0
66.005278	149.103056	1945	Dog	0
66.005278	149.103056	1945	Dog	0
66.005278	149.103056	1945	Dog	0
66.005278	149.103056	1945	Dog	0
61.859722	158.128889	1945	Red Fox	1
66.548889	152.844722	1945	Red Fox	0
66.359722	147.397500	1945	Red Fox	0
64.558056	149.090556	1945	Dog	0
64.847778	149.805000	1945	Red Fox	0
66.567500	145.256389	Jan. 1949	Dog	0
63.776111	171.700833	May. 1950	Arctic Fox	1
67.507500	148.512500	2013	Wolf	0
68.143056	151.733611	May. 1952	Wolf	0
68.143056	151.733611	May. 1952	Dog	0
68.143056	151.733611	May. 1952	Red Fox	0
68.143056	151.733611	May. 1952	Red Fox	0
68.143056	151.733611	May. 1952	Red Fox	0
60.802222	161.418611	Feb. 1954	Red Fox	1
64.503889	165.399444	Feb. 1954	Arctic Fox	1
71.295556	156.766389	Apr. 1954	Arctic Fox	1
70.647222	160.016111	Apr. 1954	Arctic Fox	1
66.897222	162.585556	Mar. 1954	Red Fox	1
66.897222	162.585556	Apr. 1954	Arctic Fox	1
63.878889	160.789722	Apr. 1954	Dog	1
66.971667	160.430278	Oct. 1954	Wolf	1
64.503889	165.399444	Mar. 1955	Red Fox	1
60.792222	161.755833	Aug. 1955	Red Fox	1
66.837222	161.036667	Oct. 1955	Red Fox	1
66.837222	161.036667	Nov. 1955	Dog	1
61.878056	162.084722	Feb. 1956	Red Fox	1
55.209167	162.714167	Feb. 1956	Red Fox	0
66.897222	162.585556	Apr. 1956	Red Fox	1

60.792222	161.755833	Apr. 1956	Dog	1
71.388889	156.479167	Sept. 1956	Arctic Fox	1
71.388889	156.479167	Oct. 1956	Arctic Fox	1
71.388889	156.479167	Oct. 1956	Arctic Fox	1
71.388889	156.479167	Oct. 1956	Arctic Fox	1
71.388889	156.479167	Oct. 1956	Arctic Fox	1
71.388889	156.479167	Oct. 1956	Arctic Fox	1
63.604167	171.204722	Jan. 1957	Arctic Fox	1
71.295556	156.766389	Feb. 1957	Arctic Fox	1
66.971667	160.430278	Mar. 1957	Arctic Fox	1
59.753333	161.902778	Nov. 1957	Red Fox	1
69.679444	144.192500	Jun. 1977	Wolf	1
69.679444	144.192500	Aug. 1977	Wolf	1
69.679444	144.192500	Aug. 1977	Wolf	1
60.792222	161.755833	1988	Red Fox	1
60.792222	161.755833	1988	Red Fox	1
60.792222	161.755833	1988	Red Fox	1
60.792222	161.755833	1988	Red Fox	1
70.132778	143.616111	1988	Red Fox	1
70.132778	143.616111	1988	Red Fox	1
60.891944	162.535833	1988	Red Fox	1
60.891944	162.535833	1988	Red Fox	1
59.440833	154.746944	1988	Red Fox	1
61.878056	162.084722	1988	Red Fox	1
64.503889	165.399444	1988	Dog	1
70.216389	151.005833	1988	Red Fox	1
70.216389	151.005833	1988	Red Fox	1
62.045278	163.218611	1988	Red Fox	1
62.045278	163.218611	1988	Red Fox	1
62.045278	163.218611	1988	Dog	1
70.647222	160.016111	1988	Red Fox	1
70.647222	160.016111	1988	Red Fox	1
67.085000	157.860278	1991	Wolf	0
71.295556	156.766389	1992 - 1993	Arctic Fox	1
71.295556	156.766389	1992 - 1993	Arctic Fox	1
71.295556	156.766389	1992 - 1993	Arctic Fox	1
71.295556	156.766389	1992 - 1993	Arctic Fox	1
71.295556	156.766389	1992 - 1993	Arctic Fox	1
71.295556	156.766389	1992 - 1993	Arctic Fox	1
60.792222	161.755833	1992 - 1993	Red Fox	1
60.792222	161.755833	1992 - 1993	Red Fox	1
64.617778	162.256667	1992 - 1993	Red Fox	1
62.777222	164.545000	1992 - 1993	Red Fox	1
66.897222	162.585556	1992 - 1993	Red Fox	1
59.451944	157.312222	1992 - 1993	Red Fox	1
64.503889	165.399444	1992 - 1993	Arctic Fox	1

64.503889	165.399444	1992 - 1993	Arctic Fox	1
64.503889	165.399444	1992 - 1993	Dog	1
70.216389	151.005833	1992 - 1993	Arctic Fox	1
68.346944	166.763056	1992 - 1993	Arctic Fox	1
68.346944	166.763056	1992 - 1993	Arctic Fox	1
68.346944	166.763056	1992 - 1993	Arctic Fox	1
68.346944	166.763056	1992 - 1993	Red Fox	1
70.325556	148.711389	1992 - 1993	Red Fox	1
59.753333	161.902778	1992 - 1993	Red Fox	1
63.471111	162.053056	1992 - 1993	Red Fox	1
63.696667	170.460833	1992 - 1993	Arctic Fox	1
66.255556	166.072222	1992 - 1993	Arctic Fox	1
69.741111	163.008611	Jan. 1997	Arctic Fox	1
69.741111	163.008611	Feb. 1997	Arctic Fox	1
63.471111	162.053056	Feb. 1997	Red Fox	1
68.346944	166.763056	Mar. 1997	Arctic Fox	1
64.355556	161.191389	Mar. 1997	Red Fox	1
64.480000	165.300000	Mar. 1997	Arctic Fox	1
67.727222	164.539167	Mar. 1997	Red Fox	1
60.792222	161.755833	Mar. 1997	Red Fox	1
61.527778	165.578611	Mar. 1997	Arctic Fox	1
62.777222	164.545000	Mar. 1997	Red Fox	1
63.471111	162.053056	Mar. 1997	Red Fox	1
61.575556	159.247222	Apr. 1997	Red Fox	1
60.693333	161.973611	Apr. 1997	Dog	1
70.325556	148.711389	Apr. 1997	Arctic Fox	1
63.035833	163.560278	Apr. 1997	Red Fox	1
60.580556	165.259444	Apr. 1997	Arctic Fox	1
60.580556	165.259444	Apr. 1997	Dog	1
59.121389	161.585833	Apr. 1997	Red Fox	1
60.792222	161.755833	Mar. 2003	Red Fox	1
62.045278	163.218611	Apr. 2003	Red Fox	1
68.071944	162.876111	Apr. 2003	Arctic Fox	1
60.792222	161.755833	Apr. 2003	Dog	1
60.342778	162.672778	Jan. 2004	Dog	1
59.953889	162.895278	Feb. 2004	Red Fox	1
61.785556	161.334167	Mar. 2004	Red Fox	1
60.891944	162.535833	Mar. 2004	Red Fox	1
62.090000	163.723889	Mar. 2004	Dog	1
61.527778	165.578611	Mar. 2004	Red Fox	1
66.897222	162.585556	Mar. 2004	Red Fox	1
61.528889	166.096111	Apr. 2004	Red Fox	1
66.897222	162.585556	Apr. 2004	Red Fox	1
65.612222	168.089167	Dec. 2005	Red Fox	1
60.693333	161.973611	Dec. 2005	Dog	1
60.693333	161.973611	Dec. 2005	Dog	1

65.758333	168.951667	Jan. 2006	Arctic Fox	1
65.612222	168.089167	Feb. 2006	Red Fox	1
66.255556	166.072222	Feb. 2006	Red Fox	1
65.612222	168.089167	Feb. 2006	Dog	1
65.612222	168.089167	Feb. 2006	Dog	1
65.612222	168.089167	Feb. 2006	Dog	1
60.944444	164.644167	Feb. 2006	Dog	1
60.792222	161.755833	2006	Dog	1
64.503889	165.399444	Feb. 2011	Arctic Fox	1
60.792222	161.755833	Mar. 2011	Red Fox	1
60.792222	161.755833	Mar. 2011	Red Fox	1
69.366944	152.144167	2012	Wolverine	1
70.208333	148.511667	1978	Caribou	1
61.216667	149.900000	Feb. 1950	Dog	0
64.843611	147.723056	1945	Red Fox	0
64.147222	145.801667	1945	Coyote	0
61.216667	149.900000	Feb. 1951	Dog	0
