# Supplementary material for: Ecological niche modeling of rabies in the changing Arctic of Alaska
Source: Acta Vet Scand. 2017 Mar 20;59:18. doi: 10.1186/s13028-017-0285-0 (PMC5359834; doi:10.1186/s13028-017-0285-0)
Supplement: Supplementary file 3 — Additional file 3. Location data of rabies cases used for model assessment. This file lists the location of rabies cases provided by the Alaska Section of Epidemiology in the Department of Health and Social Services of the State of Alaska. [file 13028_2017_285_MOESM3_ESM.pdf]

Additional file 3. Location data of rabies cases used for model assessment provided by Section of Epidemiology, Department of Health and Social Services, State of Alaska

lat	long_
59.0397	158.4575
59.9376	164.4044
59.9599	162.8903
59.9599	162.8903
60.1600	164.2658
60.1600	164.2658
60.3431	162.6631
60.4794	164.7239
60.7922	161.7558
60.7922	161.7558
60.7922	161.7558
60.7922	161.7558
60.8969	162.4594
60.8969	162.4594
60.8969	162.4594
60.8969	162.4594
61.5122	160.3581
61.5122	166.0967
61.7850	161.3203
63.6942	170.4789
63.6942	170.4789
63.6942	170.4789
63.6942	170.4789
64.5039	165.3994
64.5039	165.3994
64.6175	162.2606
64.9151	161.1569
64.9319	161.1569
65.2636	166.3608
65.7582	168.9518
65.7582	168.9519
65.7582	168.9519
65.9797	161.1231
66.0756	162.7172
66.2556	166.0722
66.6039	160.0069
66.8383	161.0328
66.8972	162.5856
66.8972	162.5856
66.8972	162.5856
66.8972	162.5856
66.8972	162.5856
67.5075	148.5125

67.7269	164.5333
67.7269	159.9988
67.7269	159.9988
68.0756	162.8561
68.0756	162.8561
68.3469	166.7631
69.7403	163.0086
69.7411	163.0086
69.7411	163.0086
70.0103	157.3994
70.0497	151.0058
70.1319	143.6239
70.1319	143.6239
70.2164	151.0058
70.4692	157.3994
70.4692	157.3994
70.4692	157.3994
70.6472	160.0153
70.6472	160.0153
70.6472	160.0161
71.2956	156.7664
71.2956	156.7664
71.2956	156.7664
71.2956	156.7664
71.2956	156.7664
71.2956	156.7664
71.2956	156.7664
71.2956	156.7664
71.2956	156.7664
71.2956	156.7664
