# Supplementary material for: Ecological niche modeling of rabies in the changing Arctic of Alaska
Source: Acta Vet Scand. 2017 Mar 20;59:18. doi: 10.1186/s13028-017-0285-0 (PMC5359834; doi:10.1186/s13028-017-0285-0)

Additional file 4. TreeNet model summary statistics for pooled rabies locations.

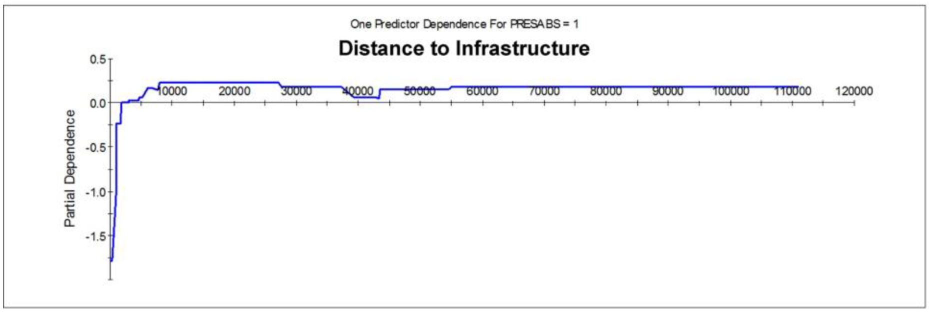

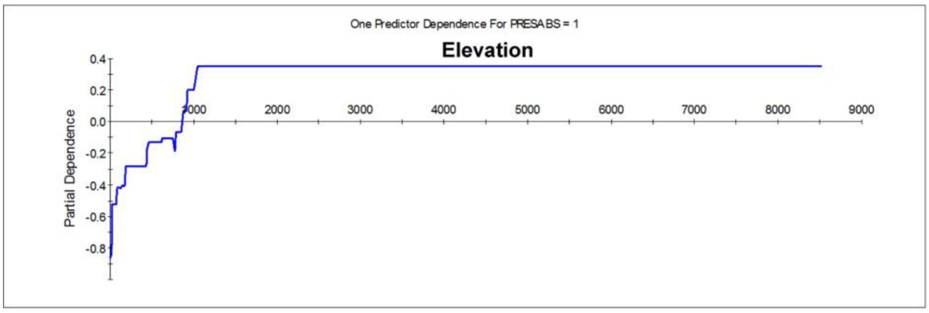

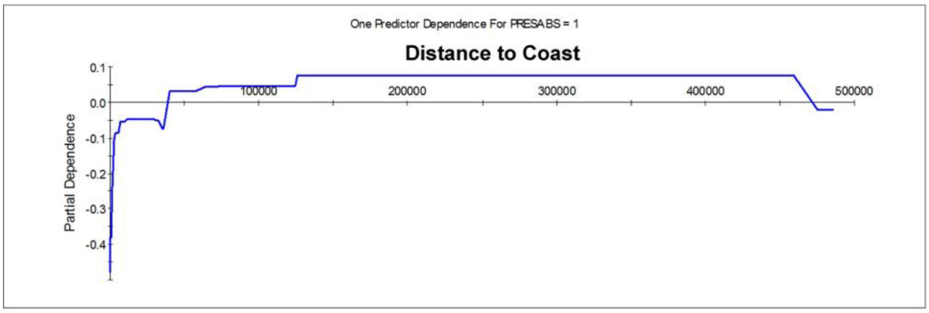

Average Log Liklihood

Summary

Name	Learn	Test
Average LogLikelihood (Negative)	0.10886	0.09594
Misclass Rate Overall (Raw)	0.02954	0.02251
ROC (Area Under Curve)	0.99486	0.99177
Lift	10.00000	9.71429
Fraction Data Used	0.22661	n/a

TreeNet Output 3: Summary For 104 Trees (Optimal) - Gains Chart - ROC, Sample: Full sample, Target class: 1

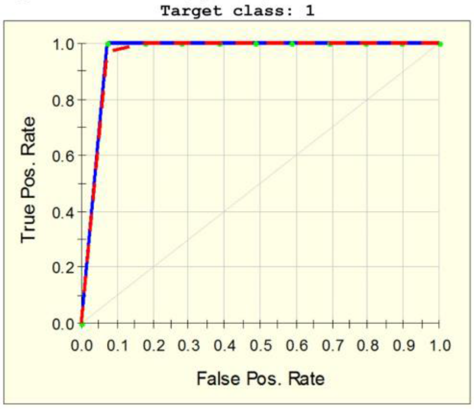

Supplement: Supplementary file 4 — Additional file 4. TreeNet model summary statistics for pooled rabies locations. This file contains summary statistics for the best performing model developed in this studies. It includes partial dependence plots for the three most important predictors in the model (distance to infrastructure, Elevation, and distance to coast), genral Average likelihood statistics and a Gains chart for 104 trees. [file 13028_2017_285_MOESM4_ESM.pdf]
